# ENGAGEMENT DURING POST–ACUTE CARE TRANSITION: PERCEPTIONS AND EXPERIENCES OF DEMENTIA FAMILY CAREGIVERS

**DOI:** 10.1093/geroni/igad104.2594

**Published:** 2023-12-21

**Authors:** Ruth Tadesse, Karen Lyons, Glenise McKenzie, Sara Hart, Lee Ellington, Kristin Cloyes

**Affiliations:** Oregon Health & Science University, Portland, Oregon, United States; Boston College, Boston, Massachusetts, United States; Oregon Health & Science University, Portland, Oregon, United States; University of Utah, Salt Lake City, Utah, United States; University of Utah, Salt Lake City, Utah, United States; Oregon Health & Science University, Portland, Oregon, United States

## Abstract

Hospitalized persons with dementia (PwD) are three times more likely to discharge to skilled nursing facilities (SNF) than those without dementia, and family caregivers are essential during post-acute care transitions (PACT). Descriptions of caregiver engagement are largely provider-centric, derived from either acute or residential settings. Little is known about PACT experiences of family caregivers. This study explored meaning, barriers, and facilitators of engagement for family caregivers navigating PACT, and elucidated caregiver support needs. An interpretive descriptive approach, using Meleis’s Middle Range Transition Theory was used to interview family caregivers during a PACT. Data were inductively coded and thematically analyzed. A conceptual framework illustrating caregivers’ meaning of engagement was developed, and facilitators, barriers, and needs were described. Participants (n=15), mostly white (n=12, 80%), female (n=13, 87%), married (n=7, 47%), adult children (n=11, 73%), average age of 63 (range: 38-81, SD=12.9). The core concept was advocacy. Key related themes were caregivers’ sense of being present, communication with providers, and meaningful connections with the PwD. Barriers to engagement centered on poor communication from professional caregivers and being isolated from the PwD. A facilitator to engagement was effective communication with professional caregivers. Similarly, caregivers’ support needs included receiving information related to the transition, and the health status of the PwD. Communication was central to both caregiver engagement and advocacy of PACT. Future policy/practice recommendations should include communication training for both providers and caregivers. Interventions that facilitate caregivers’ shared access and input into health information during PACT will promote integral roles on the care team.

